# The impact of a positive discipline group intervention on parenting self-efficacy among mothers of young children

**DOI:** 10.3389/fpubh.2024.1461435

**Published:** 2024-10-09

**Authors:** Jian Liu, Xiaojuan Liu, Meifang Ding

**Affiliations:** ^1^Department of Law School, Dongguan City University, Dongguan, China; ^2^Dongguan Children's Companion Culture Communication Consulting Co., Dongguan, China; ^3^Guangdong Teachers College of Foreign Language and Arts, Guangzhou, China

**Keywords:** positive discipline group, parenting self-efficacy, maternal intervention, diet promotion, healthy lifestyle behaviors

## Abstract

**Introduction:**

Instilling healthy behaviors in early childhood is crucial as they can have lifelong impacts. However, many Chinese mothers lack effective parenting strategies, resulting in low self-efficacy. Positive Discipline, a non-punitive and non-indulgent approach, can help enhance maternal parenting self-efficacy and promote healthy lifestyle behaviors in children. This study explores the impact of a Positive Discipline group intervention on the parenting self-efficacy of Chinese mothers and how it can contribute to promoting diet and healthy lifestyle behaviors in early childhood.

**Methods:**

We randomly selected 70 mothers with low parenting self-efficacy from a kindergarten in China, dividing them into an intervention group (35 participants) and a control group (35 participants). The intervention group received a 6-week Positive Discipline intervention, while the control group received no intervention. The intervention aimed at helping mothers nurture their children in a kind and firm manner. We used paired samples *t*-tests and independent samples *t-*tests to compare changes in parenting self-efficacy scores before and after the intervention.

**Results:**

Results showed that the intervention group's parenting self-efficacy scores significantly improved after the intervention, from an average score of 25.00 ± 4.08 to 36.29 ± 2.99 (*p* < 0.05, Cohen's d = 3.156). Significant improvements were observed in areas like “believing their parenting style benefits their child's growth” and “knowing how to effectively parent their children.” The control group's average scores showed no significant changes between pre- and post-intervention (pre: 25.07 ± 5.33; post: 24.86 ± 5.75, *p* > 0.05, Cohen's d = 0.076). Furthermore, 3 months post-intervention, the intervention group's scores remained significantly higher than pre-intervention (*p* < 0.05), demonstrating the intervention's lasting effect.

**Conclusion:**

This study demonstrates that the Positive Discipline group intervention significantly enhances parenting self-efficacy in Chinese mothers, promoting healthy lifestyle behaviors in early childhood. Providing continuous support and guidance to mothers can help solidify their parenting confidence, ensuring long-term intervention success. Future research should explore how group interventions can effectively integrate healthy behaviors into early childhood routines and impact children's diet and lifestyle behaviors.

## 1 Introduction

Parenting self-efficacy refers to parents' assessment of their ability to be effective and competent in executing parenting behaviors and their ability to positively influence their children's behavior and development. The concept is derived from self-efficacy theory, which posits that an individual's belief in their ability to accomplish specific tasks influences their actual performance and behavioral choices. Teti and Gelfand define parental self-efficacy as the application of efficacy beliefs to specific parenting domains, reflecting individuals' perceptions of their influence as parents (1). Coleman and Karraker extended the concept to mothers of young children and noted that maternal parenting efficacy refers to a mother's judgment of her competence in fulfilling her role or her perception and evaluation of her ability to positively influence her child's behavior and development (2).

With China's economic development and societal progress, educational and living standards have improved, resulting in a profound shift in parental attitudes toward parenting. The concept of high-quality parenting has gained traction, with parents increasingly emphasizing the quality of upbringing (3). However, the environment in which Chinese children grow up is filled with temptations and challenges, making traditional parenting methods such as punitive, indulgent, or neglectful approaches increasingly ineffective in modern society (4). Inappropriate parenting methods can lead to various issues in children (5). In this context, the relationship between parents' self-efficacy in parenting and the methods they use becomes especially important. When parents fail to adopt scientific parenting methods, they often feel fatigued and frustrated, which lowers their parenting self-efficacy and affects their ability to positively influence their children's behavior and development (6). This is particularly crucial during early childhood, a stage vital for children's self and character development (7). High-quality parent-child relationships, especially mother-child bonds, play a significant role in children's healthy growth and the prevention of behavioral issues (8). Research has shown that mothers of young children generally have higher parenting self-efficacy compared to fathers (9), and mothers exhibit greater sensitivity and responsiveness to their children's physiological and psychological needs (10). Therefore, understanding how mothers use positive parenting strategies, such as praise and punishment, handle children's needs, maintain patience and wisdom, and provide confidence and high-quality companionship, is crucial for the healthy development of young children (11, 12).

Positive Discipline, a non-punitive and non-indulgent approach, is grounded in Adlerian psychology. It emphasizes both kindness and firmness, aiming to cultivate responsibility, cooperation, and problem-solving skills in children. Unlike traditional parenting methods, which may focus on punishment or indulgence, Positive Discipline encourages respectful yet firm guidance, fostering a supportive environment that builds mutual respect between parents and children (13, 14). Adlerian psychology plays a critical role in Positive Discipline, promoting the idea that individuals are motivated by a desire to belong and feel significant within their community (15). In the context of parenting, this means fostering positive relationships and nurturing a sense of contribution and responsibility in children (16). Incorporating these Adlerian principles, Positive Discipline aims to empower parents with strategies that enhance their self-efficacy in managing behavior, offering a structured approach that supports both emotional and behavioral development in children (17). Research indicates that Positive Discipline training can significantly enhance parents' self-efficacy in parenting, particularly across several dimensions such as promoting health and safety, learning guidance, behavior management, social activity guidance, emotional support, and emotional regulation (18, 19). However, there is still relatively limited research on how Positive Discipline interventions impact parenting self-efficacy. Therefore, the specific objectives of this study are as follows:

(1) To evaluate the impact of Positive Discipline group intervention on improving parenting self-efficacy among Chinese mothers of young children.
(2) To explore whether the intervention has a lasting effect on mothers' parenting confidence and the promotion of healthy behaviors in early childhood.

## 2 Literature review

Scholarly research on parenting self-efficacy primarily focuses on two aspects: (1) the relationship between parental self-efficacy and child success, and (2) the current state of research on Positive Discipline interventions and parenting self-efficacy.

### 2.1 Current research on parenting self-efficacy

#### 2.1.1 Research on the relationship between parental self-efficacy and child success

Most studies investigating the relationship between parental self-efficacy and child success focus on exploring their direct relationship, while a few have introduced mediating variables to examine the interaction models among parenting style, efficacy beliefs, and child development success (20). Bandura emphasized the role of parental educational self-efficacy in child development. He pointed out that if parents believe they play an important role in their children's development and act accordingly, they will encourage their children to develop potential, help them establish intellectual efficacy and aspirations, and thus promote their emotional health, social relationships, and learning ability (21). Effective parenting also enhances personal efficacy levels in their parental role (22).

Moreover, high self-efficacy can predict positive parenting skills. Parents with high self-efficacy can provide responsive and stimulating non-punitive care (23), have high acceptance levels (24), actively interact with their children (25), tend to cope positively (26), and have a strong ability to understand infant signals (23). Conversely, low self-efficacy leads to high maternal stress (26), coercive parenting (23), negative coping strategies, anger toward children, difficult child temperament (27), and child behavior problems (28, 29). Further research shows that due to environmental and family context differences, the interaction between parental efficacy beliefs, parenting style, and child development success may vary (30, 31). In high-stress situations, efficacy beliefs have a greater impact on parenting behavior than in low-stress situations (32). Therefore, enhancing parental self-efficacy is essential for children's successful development. Interventions like Positive Discipline can help parents improve their self-efficacy, create a positive growth environment for children, and promote healthy development.

#### 2.1.2 Research on interventions in parenting self-efficacy

Intervention research on parenting self-efficacy primarily focuses on community-level interventions. By implementing various measures in community, home, or school environments, parents are encouraged to actively participate (14, 33) in parenting activities to achieve long-term effects of supportive interventions. By guiding parents on how to build positive parenting self-efficacy beliefs, these extended activities help promote children's success and development. They not only help parents believe they can influence their children's education and development, thereby building confidence in parenting but also expand and strengthen the connection between home and school in educating children, improving parental self-efficacy (34).

Chinese scholars have found in their research on the influence of parent-child relationship promotion models on maternal parenting efficacy that providing parent-child relationship education in community parenting classes helps parents understand the importance of the parent-child relationship (35) in the upbringing process. It also helps parents grasp methods for developing a good parent-child relationship, enhancing maternal confidence in parenting, improving mothers' sensitivity to children's psychological needs, and promoting the development of the parent-child relationship (36). In recent years, Positive Discipline has gradually become an important means of intervening in parental self-efficacy. The Positive Discipline method proposed by some scholars emphasizes a kind yet firm parenting style (37). By establishing cooperation, responsibility, and problem-solving abilities, it helps parents gain higher parenting confidence. Therefore, improving parents' parenting self-efficacy through intervention can positively affect children's behavior and development.

### 2.2 Research on positive discipline interventions and parenting self-efficacy

Research on Positive Discipline interventions and parental self-efficacy often focuses on home-school education and community interventions, using parenting self-efficacy as a mediator to study its effects on children's behavior and educational outcomes. McKee et al. explored the positive impact of positive parenting intervention programs on child behavior in their research (38). Feuerborn and Tyre found that both researchers and school practitioners achieved positive results from proactive practices of School-Wide Positive Behavior Support (SWPBS) (39). Jiang et al. designed and conducted a community-based intervention program (SNAP) that improved parental self-efficacy through positive intervention and reduced children's behavior problems (40). Song et al. analyzed the factors influencing parenting self-efficacy among working mothers in South Korea and suggested providing educational interventions aimed at improving mothers' role awareness and satisfaction (41).

Other scholars' research on Positive Discipline interventions mainly involves group counseling or case interventions targeting parent-child relationships (42), class management (43, 44). However, no studies directly utilize group work to conduct Positive Discipline interventions specifically focused on parental self-efficacy. As a non-punitive and non-indulgent parenting style, Positive Discipline can help parents establish positive parenting beliefs through group work, improve parenting self-efficacy (45), and cultivate cooperation, self-discipline, and responsibility in children during the educational process. Therefore, this study explores the impact of Positive Discipline group intervention on the parenting self-efficacy of Chinese mothers of young children to fill the existing research gap.

## 3 Materials and methods

### 3.1 Sample selection

This study was conducted at a public kindergarten in Dongguan. According to local education department data, the kindergarten is considered a mid-level public institution within the city, representing a typical demographic in terms of enrollment and scale. The socio-economic backgrounds of the students' families range from low to middle and high income, making the selected group of mothers representative of parenting conditions and psychological characteristics in urban areas of China, with a certain level of generalizability. Specifically, Dongguan is one of the economically developed cities in the Pearl River Delta region of China, characterized by typical urbanization and a relatively high socio-economic status. The kindergarten is located in the city center, with residents primarily from middle to high-income families, enjoying stable financial conditions. The parents in this area have a high level of education, stable employment, and diverse occupations, including white-collar workers, self-employed individuals, and technical staff, all of whom show a strong interest and active participation in parenting and education. Thus, selecting mothers from this kindergarten as the study sample ensures representation of a typical urban mother group in China, enhancing the study's relevance. Secondly, regarding the diversity of student and family backgrounds, the kindergarten enrolls both local residents and children of migrant workers admitted through a point-based system. This diversity in student sources guarantees a broad range of family, cultural, and economic backgrounds, increasing the external validity of the research findings. Particularly in Chinese cities, children of migrant worker families form a significant proportion, and selecting this kindergarten effectively represents this population, improving the generalizability of the results. Thirdly, in terms of educational quality, the kindergarten is a public institution with high standards, a teacher-to-student ratio of 1:5, and a favorable educational environment, emphasizing the holistic development of children. Researching the mothers in this context ensures that the subjects are exposed to a standardized educational framework, minimizing bias due to differences in educational environments.

### 3.2 Inclusion and exclusion criteria

**Figure 1:**
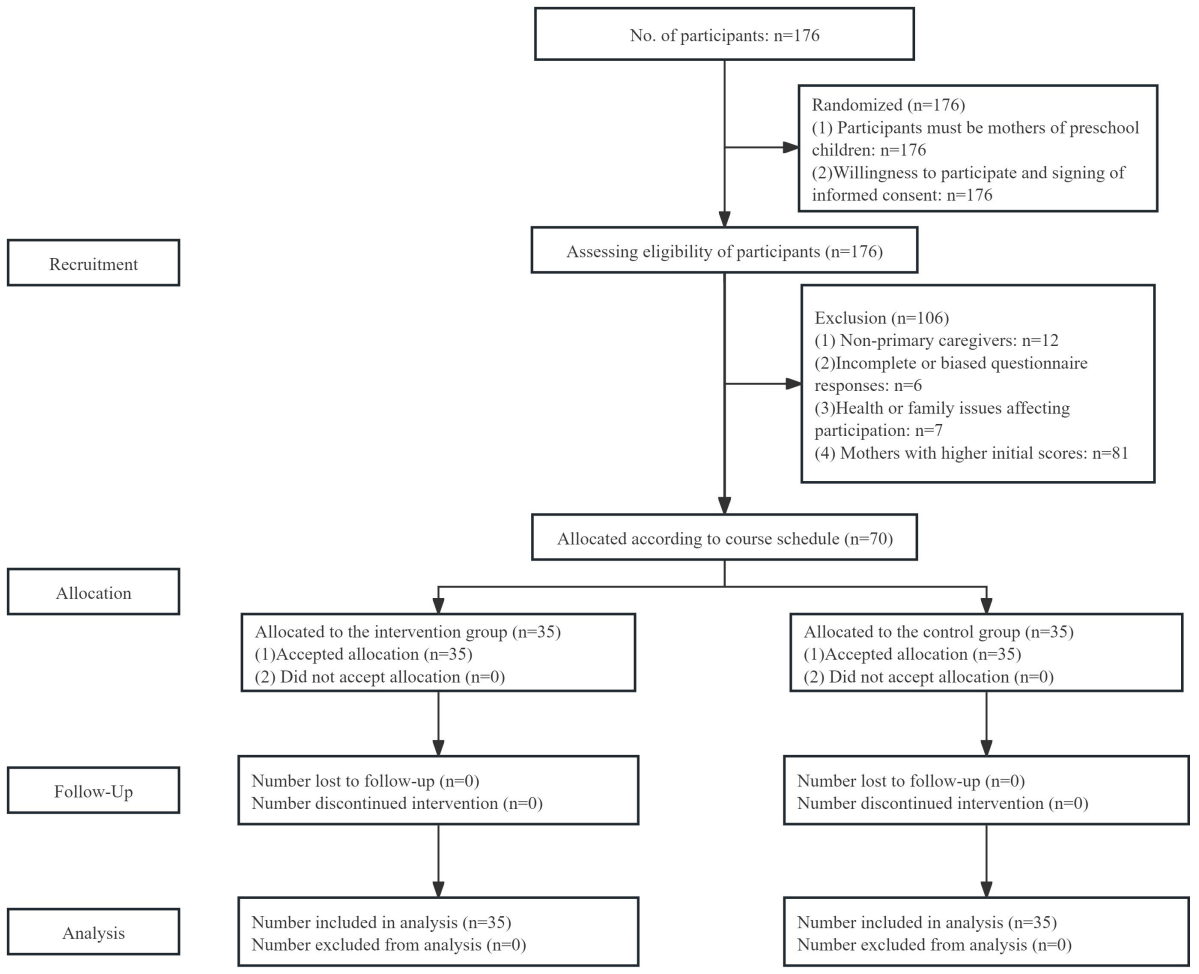
CONSORT flow diagram.

The CONSORT flowchart of this study is illustrated in Figure 1. The specific inclusion and exclusion criteria are as follows:

Inclusion Criteria:

(1) Participants must be mothers of preschool children: All participants included in the study must be mothers of children currently enrolled in kindergarten.
(2) Willingness to participate and signing of informed consent: All participants must voluntarily agree to take part in the study and sign an informed consent form after fully understanding the study's purpose, procedures, and potential risks.

Exclusion Criteria:

(1) Non-primary caregivers: If the mother is not the primary caregiver of the child (e.g., the child is mainly cared for by grandparents or other family members), her parenting self-efficacy may not accurately reflect the actual caregiving experience, and she will be excluded from the study.
(2) Incomplete or biased questionnaire responses: If there is significant missing data or logical errors in the questionnaires, either in the pre- or post-test (e.g., selecting the same answer for all questions), the data will be excluded to ensure the accuracy of the analysis.
(3) Health or family issues affecting participation: Mothers who are unable to participate in the intervention or complete the questionnaire on time due to health problems or family issues will also be excluded to ensure data validity and consistency.
(4) Mothers with higher initial scores: This study focuses on mothers with low parenting self-efficacy, so mothers who scored higher on the initial self-efficacy questionnaire will not be included in the study.

### 3.3 Tool selection

The study employed the “Parenting Self-Efficacy Scale for Mothers of Young Children,” which is based on the scale developed by Taiwanese scholar Chen Fumei. This unidimensional scale consists of 10 items, each rated on a 5-point Likert scale, with response options ranging from “not at all consistent” (1 point) to “completely consistent” (5 points). The total score is the sum of the item scores, ranging from a minimum of 10 to a maximum of 50, with higher scores indicating stronger parenting self-efficacy. In this study, the scale's reliability and validity were reassessed based on improvements made by Chinese mainland researcher Fang (46). As shown in Table 1, the scale demonstrated a Cronbach's α value of 0.847, indicating good internal consistency. The CR cutoff value, item-total correlation, and homogeneity test also met or exceeded the judgment criteria, confirming the scale's suitability for this study. After the intervention sessions, the parenting self-efficacy of mothers in both the intervention and control groups was reassessed.

**Table 1:** Test of parenting self-efficacy scale for mothers of young children.

**Item**	**CR**	**Item-total correlation**	**Homogeneity test**		
			**Cronbach's** α **after item deletion**	**Communality**	**Factor loading**
I1	5.889^***^	0.642^***^	0.832	0.586	0.688
I2	7.110^***^	0.620^***^	0.836	0.501	0.607
I3	7.759^***^	0.626^***^	0.835	0.545	0.629
I4	7.144^***^	0.633^***^	0.833	0.407	0.636
I5	7.221^***^	0.610^***^	0.836	0.444	0.611
I6	7.470^***^	0.684^***^	0.828	0.484	0.695
I7	9.563^***^	0.756^***^	0.821	0.609	0.764
I8	8.704^***^	0.656^***^	0.833	0.431	0.651
I9	9.527^***^	0.712^***^	0.825	0.619	0.711
I10	4.686^***^	0.549^***^	0.844	0.646	0.516
Judgment criteria	≥3	≥0.4	≤ 0.847	≥0.2	≥0.45

^***^represents p < 0.01.

Data analysis was performed using SPSS 17.0, beginning with descriptive statistics to summarize the demographic characteristics of the sample, such as age, education, household income, and occupation. Hypothesis testing included an independent samples *t*-test to compare parenting self-efficacy between the intervention and control groups before and after the intervention. A paired samples *t*-test was also conducted within the intervention group to evaluate changes in self-efficacy from pre- to post-intervention, thereby assessing the effectiveness of the positive discipline intervention. Statistical significance was set at *p* < 0.05, and effect sizes were calculated to determine the magnitude of the intervention's impact. These tests ensured the reliability and validity of the findings regarding the impact of the positive discipline group intervention on mothers' parenting self-efficacy.

### 3.4 Experimental intervention process

First, the researchers prepared the “Parenting Self-Efficacy Scale” in advance and numbered the questionnaires according to the children's class and student ID. For example, the questionnaire for the mother of student number 20 in class 1 was numbered 120, and the questionnaire for the mother of student number 15 in class 3 was numbered 315. Then, taking advantage of a parents' meeting at the kindergarten, paper questionnaires were distributed to the attending mothers. The researchers provided unified instructions on how to complete the questionnaires and the time requirements, ensuring that all mothers fully understood and filled out the questionnaires correctly. The questionnaires were collected immediately after completion. A total of 176 questionnaires were distributed, with 170 valid questionnaires returned, resulting in a response rate of 96.6%.

It is worth noting that, in the collected data, the highest parenting self-efficacy score among the mothers was 47, the lowest was 20, and the average score was 29. After statistical analysis, the 70 mothers with scores below the average were selected as participants for the next stage of the intervention experiment. These 70 mothers were randomly assigned to either the intervention group or the control group using a simple randomization method, with 35 mothers in each group. The intervention group participated in a 6-week positive discipline group intervention, while the control group did not receive any intervention.

The intervention design was based on Dreikurs and Nelsen's positive discipline theory, aimed at enhancing mothers' parenting self-efficacy through a series of group activities. The intervention consisted of six group sessions, each lasting 1.5 h, conducted over a 6-week period, with one session per week. The content of each session was closely related to the mothers' parenting self-efficacy, and the specific topics and procedures are shown in Table 2. All mothers in the intervention group participated in these six group activities, which were facilitated by a psychologist professionally trained in positive discipline, ensuring the scientific rigor and consistency of the intervention. Each group session included theoretical instruction, group discussions, and role-playing exercises to ensure that mothers could apply the knowledge they gained to their daily lives.

**Table 2:** Course schedule during the intervention process.

**Course number**	**Intervention topic**	**Specific intervention content**
Session 1	Introduction to positive discipline concepts	This session introduced the theoretical foundation, core principles, and implementation strategies of positive discipline, helping mothers understand the scientific and practical aspects of positive discipline.
Session 2	Learning effective communication skills	This session emphasized the importance of effective communication with children in the family setting, teaching mothers how to use positive language and non-violent communication to boost their children's confidence and enhance the mothers' self-efficacy.
Session 3	How to effectively encourage children	This session explored the importance and methods of encouraging children, teaching mothers how to use encouragement to foster their children's self-worth and, in turn, enhance their own parenting self-efficacy.
Session 4	Solving specific parenting issues	This session involved case analysis and role-playing to address the real-life parenting challenges that mothers encounter, helping them apply positive discipline strategies to manage their children's behavior.
Session 5	Emotional management in positive discipline	This session focused on strategies for managing mothers' emotions during the parenting process, teaching them how to regulate their own emotions and build confidence when facing challenges.
Session 6	Review and reflection	This session reviewed the content learned in the previous sessions, allowing mothers to share personal experiences and discuss how they could continue applying positive discipline concepts to further enhance their parenting self-efficacy.

The mothers in the control group did not receive any form of intervention during the study. The kindergarten did not offer regular parenting training or intervention programs, so the control group mothers only participated in the pre- and post-measurements of parenting self-efficacy, ensuring that the only difference between the experimental and control groups was the participation in the intervention activities.

## 4 Results

### 4.1 Analysis of the demographic characteristics of the sample

The demographic characteristics of the sample reveal that all 70 participants were female, with 35 in the intervention group and 35 in the control group. The majority (49) had urban household registration, while 21 were from rural areas. In terms of age, most participants were between 30–40 years old (37), followed by 20–30 years old (29), and only 4 participants were aged 40–50. Education levels were relatively high, with 21 holding postgraduate degrees, 36 having normal course education, and 13 having completed senior secondary school. Regarding household income, the majority earned between 10,000–50,000 RMB monthly, with 14 earning over 50,000 RMB. Occupational distribution showed that the largest group (22) worked in social production and service roles, followed by clerks (15), professional and technical staff (11), and leaders of local government agencies, party organizations, and enterprises (8). Other occupations were more varied, with smaller numbers in agriculture, forestry, and fishery, transportation equipment operation, military service, and other non-classifiable professions (Table 3).

**Table 3:** Demographic characteristics of the sample.

**Variable name**	**Group**	**Number of people**
Sample size	Intervention group	35
	Control group	35
Gender	Male	0
	Female	70
Household registration type	Rural	21
	Urban	49
Age	20–30	29
	30–40	37
	40–50	4
Degree	Postgraduates	21
	Normal courses	36
	Senior secondary schools	13
	Junior secondary schools	0
	Primary schools	0
Household monthly income (RMB)	>50,000	14
	30,000–50,000	16
	10,000–30,000	22
	< 10,000	18
Occupational types of mothers of young children	Leaders of local government agencies, party and mass organizations, enterprises, and public institutions	8
	Professional and technical staff	11
	Clerks and related personnel	15
	Social production and service personnel	22
	Production and auxiliary personnel in agriculture, forestry, animal husbandry, and fishery	5
	Production and transportation equipment operators and related personnel	6
	Military personnel	1
	Other professionals not easily classified	2

### 4.2 Comparison of pre- and post-intervention scores in the group

#### 4.2.1 Comparison of pre-intervention scores between the intervention and control groups

Table 4 shows the results of independent samples *t*-tests conducted on the pre-intervention total scores and individual item scores of parenting self-efficacy for the intervention and control groups. The results indicate that the mean scores between the intervention and control groups showed no significant differences in the total score or individual item scores (*p* > 0.05). This suggests that before the group intervention, the total scores and individual item scores of parenting self-efficacy in the intervention and control groups were similar, with no significant differences.

**Table 4:** Comparison of pre-intervention parenting self-efficacy scores between the intervention and control groups.

**Parenting self-efficacy**	**Group**	***M***	***SD***	***t***	***P***	***Cohen's d***
I believe my discipline methods contribute to my child's growth.	Control group	2.86	0.77	1.325	0.197	0.055
	Intervention group	2.5	0.65			
I believe I can correct the inappropriate concepts my child learns from society.	Control group	3.29	0.994	1.832	0.078	0.073
	Intervention group	2.71	0.611			
I am confident in my parenting methods.	Control group	2.5	0.76	−0.589	0.561	0.021
	Intervention group	2.64	0.497			
I know how to effectively parent my child.	Control group	2.21	0.579	−0.288	0.776	0.122
	Intervention group	2.29	0.726			
I am satisfied with my ability to parent my child.	Control group	2.43	0.852	−0.249	0.805	0.092
	Intervention group	2.5	0.65			
I believe I am a good mother.	Control group	2.71	0.825	0.24	0.812	0.089
	Intervention group	2.64	0.745			
I feel that my parenting skills are not inferior to others.	Control group	2.29	0.825	−1.689	0.103	0.063
	Intervention group	2.71	0.469			
Compared to other parents, I know more about motherhood.	Control group	2.5	0.855	0	1	0.004
	Intervention group	2.5	0.65			
I know how to play the role of a good mother.	Control group	2.36	0.745	−0.502	0.62	0.126
	Intervention group	2.5	0.76			
I believe I am qualified to offer parenting advice to other mothers.	Control group	1.93	0.73	−0.234	0.817	0.087
	Intervention group	2	0.877			
Total score of the scale	Control group	25.07	5.327	0.04	0.969	0.015
	Intervention group	25	4.076			

For example, the mean score for the item “I believe my discipline methods contribute to my child's growth” was 2.50 in the intervention group and 2.86 in the control group, with a small Cohen's d of 0.055, indicating minimal practical difference. Similarly, for the item “I believe I can correct the inappropriate concepts my child learns from society,” the control group had a higher mean (3.29) compared to the intervention group (2.71), but this difference was also not statistically significant, with a Cohen's d of 0.073, reflecting a small effect size. Other items, such as “I am confident in my parenting methods” and “I am satisfied with my ability to parent my child,” had minimal differences between the two groups, with Cohen's d values of 0.021 and 0.092, respectively, again suggesting no meaningful pre-intervention differences. The total score of parenting self-efficacy was nearly identical between the two groups (25.07 for the control group and 25.00 for the intervention group), resulting in an extremely small Cohen's d of 0.015. These results confirm that the intervention and control groups were well-matched in terms of parenting self-efficacy before the intervention began, providing a solid foundation for comparing post-intervention changes.

#### 4.2.2 Comparison of pre- and post-intervention scores in the intervention group

Table 5 shows the results of paired samples *t-*tests comparing the pre- and post-intervention mean scores of parenting self-efficacy total and individual item scores in the intervention group. The results indicate that, overall, the total score of parenting self-efficacy after the intervention is significantly higher than before the intervention (*p* < 0.05), with the total score increasing from 25.00 to 36.29. Cohen's d for the total score was 3.156, indicating a very large effect size. Similarly, the post-intervention scores for individual items are also significantly higher than the pre-intervention scores (*p* < 0.05), with large effect sizes ranging from 1.394 to 2.776, showing a substantial improvement in various dimensions of parenting self-efficacy. For example, the mean score for the belief that “I believe my discipline methods contribute to my child's growth” increased from 2.50 to 3.93, with a large effect size of Cohen's d = 2.512. Similarly, the confidence in correcting inappropriate concepts learned from society improved from 2.71 to 3.64, with an effect size of Cohen's d = 1.495, indicating a significant impact. Other items, such as “I know how to effectively parent my child,” showed a notable increase in mean scores from 2.29 to 4.00, with an effect size of Cohen's d = 2.646, emphasizing the intervention's strong influence. These results demonstrate that the Positive Discipline intervention had a profound effect on improving various aspects of parenting self-efficacy among mothers of young children, making them more confident in their parenting abilities and better equipped to manage parenting challenges.

**Table 5:** Comparison of pre- and post-intervention parenting self-efficacy scores in the intervention group.

**Parenting self-efficacy**	**Pre-test**		**Post-test**		***t***	***P***	***Cohen's d***
	***M***	***SD***	***M***	***SD***			
I believe my discipline methods contribute to my child's growth.	2.50	0.650	3.93	0.475	−8.272	0.000	2.512
I believe I can correct the inappropriate concepts my child learns from society.	2.71	0.611	3.64	0.633	−5.643	0.000	1.495
I am confident in my parenting methods.	2.64	0.497	3.43	0.514	−4.204	0.001	1.563
I know how to effectively parent my child.	2.29	0.726	4.00	0.555	−10.494	0.000	2.646
I am satisfied with my ability to parent my child.	2.50	0.650	3.29	0.469	−5.078	0.000	1.394
I believe I am a good mother.	2.64	0.745	3.79	0.802	−6.450	0.000	1.486
I feel that my parenting skills are not inferior to others.	2.71	0.469	3.43	0.514	−5.701	0.000	1.463
Compared to other parents, I know more about motherhood.	2.50	0.650	3.29	0.469	−4.204	0.001	1.394
I know how to play the role of a good mother.	2.50	0.760	4.21	0.426	−8.832	0.000	2.776
I believe I am qualified to offer parenting advice to other mothers.	2.00	0.877	3.29	0.469	−4.837	0.000	1.834
Total score of the scale	25.00	36.29	4.076	2.998	−11.931	0.000	3.156

#### 4.2.3 Comparison of pre- and post-intervention scores in the control group

Table 6 shows the results of paired samples *t-*tests comparing the pre- and post-intervention mean scores of parenting self-efficacy total and individual item scores in the control group. The results indicate no significant differences in the mean scores between the pre- and post-intervention total scores or individual item scores (*p* > 0.05). This suggests that without the group work intervention, the parenting self-efficacy levels of the control group remain largely unchanged over time. For example, the mean score for the belief that “I believe my discipline methods contribute to my child's growth” increased only marginally from 2.86 to 2.93, with a very small effect size of Cohen's d = 0.087, indicating minimal impact. Similarly, the mean score for 'I am confident in my parenting methods' changed slightly from 2.50 to 2.64, with a small effect size of Cohen's d = 0.069, further supporting the conclusion that there were no significant improvements in parenting self-efficacy in the control group. Additionally, for the item “I believe I can correct the inappropriate concepts my child learns from society,” the mean score dropped from 3.29 to 3.07, but with a small effect size (Cohen's d = 0.109), indicating an insignificant change. Similarly, the total parenting self-efficacy score decreased slightly from 25.07 to 24.86, with a Cohen's d of 0.076, confirming the overall lack of change in the control group's parenting self-efficacy over time.

**Table 6:** Comparison of pre- and post-intervention parenting self-efficacy scores in the control group.

**Parenting self-efficacy**	**Pre-test**		**Post-test**		***t***	***P***	***Cohen's d***
	***M***	***D***	***M***	***SD***			
I believe my discipline methods contribute to my child's growth.	2.86	0.770	2.93	0.829	−0.563	0.583	0.087
I believe I can correct the inappropriate concepts my child learns from society.	3.29	0.994	3.07	0.616	1.147	0.272	0.109
I am confident in my parenting methods.	2.50	0.760	2.64	0.633	−1.000	0.336	0.069
I know how to effectively parent my child.	2.21	0.579	2.21	0.802	0.000	1.000	0.007
I am satisfied with my ability to parent my child.	2.43	0.852	2.36	0.842	0.434	0.671	0.118
I believe I am a good mother.	2.71	0.825	2.57	0.852	1.472	0.165	0.122
I feel that my parenting skills are not inferior to others.	2.29	0.825	2.14	0.663	1.000	0.336	0.080
Compared to other parents, I know more about motherhood.	2.50	0.855	2.21	0.699	1.749	0.104	0.134
I know how to play the role of a good mother.	2.36	0.745	2.50	0.650	−0.618	0.547	0.109
I believe I am qualified to offer parenting advice to other mothers.	1.93	0.730	1.93	0.616	0.000	1.000	0.103
Total score of the scale	25.07	24.86	5.327	5.749	0.295	0.773	0.076

#### 4.2.4 Comparison of post-intervention scores between the intervention and control groups

Table 7 shows the results of independent samples *t*-tests comparing the post-intervention mean scores of parenting self-efficacy total and individual item scores in the intervention and control groups. The results indicate that the post-intervention mean scores for both the total score and individual items in the intervention group are significantly lower than those in the control group (*p* < 0.05). For example, the mean score for “I believe my discipline methods contribute to my child's growth” was 2.93 in the intervention group compared to 3.93 in the control group, with a large effect size of Cohen's d = 1.027, indicating a substantial difference in this specific dimension. Similarly, for the belief that “I know how to effectively parent my child,” the control group scored significantly higher (M = 4.00) than the intervention group (M = 2.21), with Cohen's d = 1.786, showing a strong effect size. Effect sizes for most items ranged from moderate to large, such as for “I am confident in my parenting methods” (Cohen's d = 0.786), “I believe I am a good mother” (Cohen's d = 1.214), and “I feel that my parenting skills are not inferior to others” (Cohen's d = 1.286). These results suggest that the Positive Discipline intervention was effective in significantly enhancing the parenting self-efficacy of mothers in the intervention group compared to the control group, leading to substantial improvements in their confidence and abilities as parents.

**Table 7:** Comparison of post-intervention parenting self-efficacy scores between the intervention and control groups.

**Parenting self-efficacy**	**Group**	***M***	**Mean difference**	***t***	***P***	***Cohen's d***
I believe my discipline methods contribute to my child's growth.	Control group	3.93	1	2.039	0.001	1.027
	Intervention group	2.93				
I believe I can correct the inappropriate concepts my child learns from society.	Control group	3.64	0.571	1.16	0.023	0.571
	Intervention group	3.07				
I am confident in my parenting methods.	Control group	3.43	0.786	0.046	0.001	0.786
	Intervention group	2.64				
I know how to effectively parent my child.	Control group	4	1.786	5.625	0	1.786
	Intervention group	2.21				
I am satisfied with my ability to parent my child.	Control group	3.29	0.929	4.955	0.001	0.929
	Intervention group	2.36				
I believe I am a good mother.	Control group	3.79	1.214	0.071	0.001	1.214
	Intervention group	2.57				
I feel that my parenting skills are not inferior to others.	Control group	3.43	1.286	0	0	1.286
	Intervention group	2.14				
Compared to other parents, I know more about motherhood.	Control group	3.29	1.071	1.725	0	1.071
	Intervention group	2.21				
I know how to play the role of a good mother.	Control group	4.21	1.714	5.903	0	1.714
	Intervention group	2.5				
I believe I am qualified to offer parenting advice to other mothers.	Control group	3.29	1.357	0.006	0	1.357
	Intervention group	1.93				
Total score of the scale	Control group	36.29	11.429	4.267	0	2.429
	Intervention group	24.86				

#### 4.2.5 Comparison of 3-month post-intervention and pre-intervention scores in the intervention group

To verify the sustained intervention effect of the Positive Discipline group, a follow-up assessment was conducted on the intervention group 3 months after the sessions ended. Table 8 shows that the total score of parenting self-efficacy 3 months after the intervention remains significantly higher than the pre-intervention score (*p* < 0.05), with a large effect size (Cohen's d = 2.853). This indicates that the positive effects of the intervention are sustained over time. Individual items also reflect significant improvements. For instance, the mean score for “I believe my discipline methods contribute to my child's growth” increased from 2.50 to 3.50, with a large Cohen's d = 2.404, indicating a strong and lasting effect. Similarly, “I believe I can correct the inappropriate concepts my child learns from society” showed a substantial improvement, with a mean increase from 2.71 to 3.79 and Cohen's d = 2.900. Notably, items like “I know how to effectively parent my child” and “I know how to play the role of a good mother” also exhibited significant gains, with Cohen's d values of 1.798 and 2.961, respectively, highlighting a robust and lasting impact on these dimensions of parenting self-efficacy (Table 8). For these items, mothers continue to feel more confident in their parenting skills even months after the group sessions concluded.

**Table 8:** Comparison of 3-month post-intervention and pre-intervention parenting self-efficacy scores in the intervention group.

**Parenting self-efficacy**	**Pre-test**		**3-Month post-test**		***t***	***P***	***Cohen's d***
	***M***	***SD***	***M***	***SD***			
I believe my discipline methods contribute to my child's growth.	2.50	0.650	3.50	0.519	−5.508	0.000	2.404
I believe I can correct the inappropriate concepts my child learns from society.	2.71	0.611	3.79	0.426	−6.511	0.000	2.900
I am confident in my parenting methods.	2.64	0.497	3.07	0.475	−3.122	0.008	1.251
I know how to effectively parent my child.	2.29	0.726	3.07	0.475	−4.204	0.001	1.798
I am satisfied with my ability to parent my child.	2.50	0.650	3.07	0.267	−3.309	0.006	1.622
I believe I am a good mother.	2.64	0.745	3.36	0.633	−4.372	0.001	1.473
I feel that my parenting skills are not inferior to others.	2.71	0.469	2.86	0.363	−1.000	0.336	0.136
Compared to other parents, I know more about motherhood.	2.50	0.650	2.93	0.267	−2.121	0.054	0.224
I know how to play the role of a good mother.	2.50	0.760	3.79	0.426	−6.624	0.000	2.961
I believe I am qualified to offer parenting advice to other mothers.	2.00	0.877	2.71	0.611	−2.687	0.019	1.329
Total score of the scale	25.00	4.076	32.14	2.905	−6.501	0.000	2.853

However, for the items “I feel that my parenting skills are not inferior to others” and “Compared to other parents, I know more about motherhood,” the differences were not statistically significant (*p* > 0.05), with lower effect sizes (Cohen's d = 0.136 and 0.224, respectively). This suggests that while the intervention had a lasting effect on most aspects of parenting self-efficacy, these particular dimensions saw a slight decrease over time, returning closer to pre-intervention levels. Overall, the follow-up results indicate that the Positive Discipline group work leads to significant and sustained improvements in parenting self-efficacy, particularly in areas like confidence in parenting methods and the belief that one's parenting style benefits the child's growth.

#### 4.2.6 Comparison of 3-month post-intervention and pre-intervention scores in the control group

Table 9 shows the results of paired samples *t*-tests comparing the pre-intervention and 3-month post-intervention mean scores of parenting self-efficacy total and individual item scores in the control group. The results indicate that there are no significant differences in the mean scores between the pre- and 3-month post-intervention total scores or individual item scores (*p* > 0.05). This further suggests that without group work intervention, the parenting self-efficacy levels of the control group do not change over time (*p* > 0.05), meaning that the parenting self-efficacy of the control group remains largely unchanged.

**Table 9:** Comparison of 3-month post-intervention and pre-intervention parenting self-efficacy scores in the control group.

**Parenting self-efficacy**	**Pre-test**		**3-Month post-test**		***t***	***P***	***Cohen's d***
	***M***	***SD***	***M***	***SD***			
I believe my discipline methods contribute to my child's growth.	2.86	0.770	2.79	0.802	1.000	0.336	0.126
I believe I can correct the inappropriate concepts my child learns from society.	3.29	0.994	3.21	0.699	0.291	0.775	0.132
I am confident in my parenting methods.	2.50	0.760	2.71	0.726	−1.385	0.189	0.043
I know how to effectively parent my child.	2.21	0.579	2.29	0.726	−0.434	0.671	0.172
I am satisfied with my ability to parent my child.	2.43	0.852	2.29	0.726	0.806	0.435	0.150
I believe I am a good mother.	2.71	0.825	2.64	0.633	0.563	0.583	0.135
I feel that my parenting skills are not inferior to others.	2.29	0.825	2.14	0.663	1.000	0.336	0.183
Compared to other parents, I know more about motherhood.	2.50	0.855	2.21	0.699	1.295	0.218	0.125
I know how to play the role of a good mother.	2.36	0.745	2.50	0.650	−1.000	0.336	0.283
I believe I am qualified to offer parenting advice to other mothers.	1.93	0.730	2.07	0.730	−0.618	0.547	0.071
Total score of the scale	25.07	5.327	24.93	5.850	0.231	0.821	0.035

Table 9 shows the results of paired samples *t*-tests comparing the pre-intervention and 3-month post-intervention mean scores of parenting self-efficacy total and individual item scores in the control group. The results indicate that there are no significant differences in the mean scores between the pre- and post-intervention total scores or individual item scores (*p* > 0.05, suggesting that without the Positive Discipline group intervention, the parenting self-efficacy levels of the control group do not change over time. The total score of parenting self-efficacy changed only slightly from 25.07 to 24.93, with a negligible effect size (Cohen's d = 0.035). For individual items, such as “I believe my discipline methods contribute to my child's growth,” the mean score decreased marginally from 2.86 to 2.79, with a small effect size (Cohen's d = 0.126), indicating minimal change. Similarly, for “I believe I can correct the inappropriate concepts my child learns from society,” the mean score showed a small decrease from 3.29 to 3.21, with an effect size of Cohen's d = 0.132, reflecting no significant difference. Other items, like “I know how to effectively parent my child,” showed slight improvements in the post-test (from 2.21 to 2.29), but these changes were not statistically significant, with a small Cohen's d of 0.172. Even in areas where there were slight improvements or decreases, such as “I am satisfied with my ability to parent my child” (pre-test: 2.43, post-test: 2.29, Cohen's d = 0.150), the effect sizes remained small and non-significant, indicating that without intervention, the parenting self-efficacy of the control group remained largely stable over time.

## 5 Discussion

### 5.1 Discussion on the comparison of pre-intervention scores between the intervention and control groups

The independent samples *t-*test results revealed no significant differences in the pre-intervention total scores or individual item scores of parenting self-efficacy between the intervention and control groups. This suggests that before the Positive Discipline group intervention, both groups were comparable in terms of parenting self-efficacy. The homogeneity observed in the groups is critical, as it ensures that any differences observed after the intervention can be attributed to the intervention itself, rather than pre-existing disparities between the two groups. This homogeneity is supported by the demographic data collected before the intervention, which showed no significant differences in parenting self-efficacy based on the mothers' age, number of children, education level, or average monthly family income. The randomization process and independent *t-*tests further confirmed that these variables were evenly distributed across both groups. This alignment in demographic variables strengthens the study's internal validity by ensuring that the intervention and control groups were starting from the same baseline in terms of their parenting self-efficacy. Additionally, the lack of significant differences in prior exposure to Positive Discipline methods between the two groups ensured that both started with similar levels of familiarity and experience, making post-intervention comparisons more reliable.

These findings are consistent with prior research. For example, Coleman and Karraker (2) found that parenting self-efficacy did not vary significantly across demographic variables such as age and education level, while Dumka et al. also reported that factors like the number of children or mothers' education levels had minimal impact on parenting self-efficacy (24). Such consistency with established literature reinforces the validity of the study's findings, indicating that the random assignment and demographic homogeneity provided a solid foundation for evaluating the impact of the Positive Discipline intervention. By ensuring that the groups were comparable in both demographic variables and initial parenting self-efficacy, this study provides a robust basis for examining the effects of the Positive Discipline group intervention. The observed improvements in parenting self-efficacy in the intervention group can thus be confidently attributed to the intervention itself, as the control group, which did not receive the intervention, showed no significant changes in parenting self-efficacy over time. This methodological rigor enhances the credibility of the study's findings and supports the effectiveness of the Positive Discipline intervention in improving parenting self-efficacy.

### 5.2 Discussion on the comparison of pre- and post-intervention scores in the intervention group

The paired samples *t-*test results showed that post-intervention scores were significantly higher than pre-intervention scores for both the overall parenting self-efficacy and individual items in the intervention group. This indicates that the Positive Discipline group intervention effectively enhanced the parenting self-efficacy of mothers with young children, particularly by improving their ability to become more competent and effective in influencing their children's behavior and development. Specifically, the intervention led to notable improvements in areas such as confidence in managing behavior, guiding learning, and fostering emotional connections with their children. These findings align with those of Pan and Xi (19), who reported that Positive Discipline interventions significantly increased parental efficacy in multiple areas, including health and safety promotion, behavior management, and emotional care.

One of the primary reasons for the success of the intervention lies in the theoretical foundation of Positive Discipline, which is grounded in Adlerian psychology. Positive Discipline emphasizes the importance of respecting children's physical and emotional development while maintaining firm boundaries, which fosters both warmth and structure in parenting (47). This dual focus on kindness and firmness is particularly effective in building parents' confidence, as it provides clear guidelines for addressing children's needs without resorting to punitive measures. The practical tools provided during the intervention gave mothers concrete strategies for real-life application, which contributed to their increased self-efficacy.

Moreover, the interactive nature of the Positive Discipline sessions—featuring scenario experiences, brainstorming, peer support, and role-playing—draws heavily on Bandura's four sources of self-efficacy: mastery experiences, vicarious learning, verbal persuasion, and emotional arousal. By allowing participants to practice new skills in a supportive environment, the intervention not only enhanced their self-efficacy but also encouraged long-term behavior change. These group-based elements created opportunities for mothers to observe and learn from each other, fostering successful vicarious experiences and a sense of competence. In addition, the ongoing engagement promoted by the researchers, both during and after the intervention sessions, ensured sustained participation and consistent use of Positive Discipline strategies in real-life contexts. The researchers' continuous support, through support, through both pre-session invitations and post-session interviews, reinforced the intervention's impact, ensuring that participants could implement what they learned effectively. This ongoing reinforcement is critical, as it helps participants transition from learning new skills to consistently applying them in everyday parenting situations, thereby contributing to the significant improvements in parenting self-efficacy.

Overall, the Positive Discipline group intervention not only increased the participants' confidence in in their parenting abilities but also equipped them with practical tools and strategies to maintain this confidence over time. The structured support, combined with the practical application of principles, resulted in significant improvements in both overall parenting self-efficacy and specific parenting practices.

### 5.3 Discussion on the comparison of pre- and post-intervention, and 3-month post-intervention scores in the control group

The paired samples *t-*test results revealed no significant differences in the overall and individual item scores of parenting self-efficacy between the pre- and post-intervention assessments in the control group. This suggests that without the benefit of any intervention, the parenting self-efficacy of mothers with young children remains unchanged over time. Specifically, their confidence in their ability to effectively manage their children's behavior, support their development, and act as competent mothers did not improve. This finding underscores the importance of structured interventions, such as Positive Discipline group work, for enhancing parental self-efficacy. Without such interventions, parents may lack the tools and reinforcement needed to boost their confidence and parenting skills.

This conclusion aligns with Fang's (46) study on the relationship between social support in family education and parenting efficacy, which found that mothers who receive more companionship, informational, emotional, and instrumental support tend to have stronger parenting efficacy. Among these types of support, companionship and emotional support demonstrated particularly strong positive correlations with increased self-efficacy. The absence of these forms of support in the control group could explain the lack of improvement in their parenting self-efficacy. Fang's findings highlight how critical external reinforcement is to building and maintaining parental confidence. The findings of Açikgöz and Yoruk (48) and Lutfiani et al. (49) further support this conclusion, as both studies indicate that when mothers do not receive external support in the form of educational interventions or community assistance, their parenting self-efficacy remains relatively stable. Parenting self-efficacy is shaped by prolonged experiences and is relatively resistant to change without targeted external influences. In the control group, mothers continued to rely on pre-existing parenting knowledge and strategies, which had not been bolstered by new information, training, or support. Consequently, their self-efficacy levels remained at baseline, with no significant improvements observed.

This highlights the necessity of providing continuous external support or structured interventions to enable mothers to enhance their parenting self-efficacy over time. Without intervention, their perceptions of their parenting abilities and their confidence in positively influencing their children's development remain unchanged. This suggests that parenting self-efficacy is not self-reinforcing and requires active support through social or educational interventions to foster significant growth.

### 5.4 Discussion on the comparison of post-intervention scores between the intervention and control groups

The independent samples *t-*test results revealed that the post-intervention mean scores of parenting self-efficacy in the intervention group were significantly higher than those in the control group. This finding indicates that the Positive Discipline group intervention had a substantial and lasting impact on improving parenting self-efficacy for mothers with young children, whereas those who did not receive the intervention showed no such improvement. The success of the intervention can be attributed to several key factors. First, the Positive Discipline principles, which focus on a balance of kindness and firmness, allowed mothers to better understand their children's fundamental needs while learning practical strategies to manage behavior effectively. The tools and techniques provided during the sessions created opportunities for vicarious learning, allowing participants to observe successful practices and apply them in their own lives. Additionally, group interaction and experiential activities, such as role-playing and discussions, contributed to enhanced self-efficacy through verbal persuasion and mutual support (50). These group dynamics provided mothers with the confidence and encouragement to try new approaches and make positive changes in their parenting. In contrast, the control group, lacking these structured opportunities for skill-building and reinforcement, did not experience similar gains. Without intervention, the control group mothers missed out on the sources of enhanced parenting self-efficacy, such as physiological arousal, vicarious learning, and successful experiences, which explains the absence of significant improvement in their parenting abilities over time.

### 5.5 Discussion on the comparison of 3-month post-intervention and pre-intervention scores in the intervention group

The paired samples *t-*test results comparing the pre-intervention and 3-month post-intervention scores revealed that the total parenting self-efficacy score in the intervention group remained significantly higher 3 months after the Positive Discipline group intervention ended. For most individual items, eight out of ten showed significant improvements compared to pre-intervention scores. However, for the items “I feel that my parenting skills are on par with others” and “Compared to other parents, I know more about motherhood,” no significant changes were observed. These findings suggest that the Positive Discipline group intervention has a lasting impact on improving parenting self-efficacy, but certain areas may require additional reinforcement over time. The sustained improvements can be attributed to the supportive and structured nature of the Positive Discipline principles, which provided mothers with practical tools to boost their parenting self-efficacy. Throughout the intervention, the group format encouraged active participation, mutual support, and consistent application of the methods learned. The researchers also helped maintain high efficacy levels by conducting follow-up interviews and ensuring that participants continued to apply Positive Discipline strategies in their daily lives.

However, as time passes after the group sessions end, some mothers may experience a decline in their efficacy, particularly in areas where long-term practice and reflection are required to fully internalize new habits (51, 52). Without ongoing reinforcement, some aspects of parenting self-efficacy may return to levels closer to those observed before the intervention. This indicates that, while group interventions are effective in the short term, sustained improvements in parenting self-efficacy require continued support and practice to solidify the gains and prevent regression (53). Therefore, long-term supportive measures are essential for maintaining the positive effects of the intervention and encouraging the internalization of effective parenting beliefs and practices.

## 6 Conclusions

This study demonstrates that Positive Discipline group intervention has a significant and lasting impact on improving the parenting self-efficacy of Chinese mothers with young children. The intervention notably enhanced the overall parenting confidence of mothers, reflected in the significant improvement in total parenting self-efficacy scores and across all dimensions, compared to both pre-intervention levels and the control group. These findings highlight the effectiveness of Positive Discipline in helping mothers develop scientific parenting skills, establish positive parenting beliefs, and boost their confidence in handling child-rearing challenges. The positive effects of the intervention were sustained 3 months after the program concluded, further underscoring its lasting impact. Although most aspects of parenting self-efficacy remained high post-intervention, slight decreases were observed in areas such as “feeling that my parenting skills are on par with others” and “knowing more about motherhood compared to other parents.” This suggests that continued support may be necessary to maintain and strengthen these dimensions over time.

In addition, the study highlighted that Positive Discipline group intervention contributes to fostering healthy behaviors in both mothers and their children. Themes like “Positive Language,” “Emotional Management,” and “Managing Misbehavior” were instrumental in helping mothers promote healthy eating habits, safety, emotional regulation, and positive behavior in their children. The improvement in these areas suggests that parenting self-efficacy plays a critical role in shaping children's healthy behavioral development.

Based on the findings of this study, several strategies are recommended to further enhance the effectiveness of Positive Discipline interventions. First, it is crucial to provide continuous post-intervention support, such as follow-up sessions or peer networks, to reinforce the skills learned and maintain improvements in parenting self-efficacy over time. Second, interventions should be tailored to the diverse backgrounds of mothers, ensuring that content is adapted to meet the unique needs of participants from different social, economic, and cultural contexts. This would make the program more inclusive and effective. Third, integrating Positive Discipline principles into daily routines and collaborating with community resources such as schools and local organizations can help mothers apply the strategies in real-life situations, providing ongoing support and promoting healthy family dynamics. These measures will contribute to more sustainable and impactful outcomes for both mothers and their children.

## 7 Limitations

This study mainly focused on the significance level of differences in parenting self-efficacy of the research subjects after participating in six Positive Discipline group sessions, and comparing the results 3 months after the sessions ended with the pre-test results. The study covered a relatively short period, while efficacy is a relatively stable intrinsic perception and evaluation formed through long-term experience. Due to the inability of this study to examine the impact of the Positive Discipline group on parenting self-efficacy over a longer time span, future research could further explore the influence of time factors after group intervention on parenting self-efficacy to assess the long-term, sustained impact of Positive Discipline group intervention on enhancing parenting self-efficacy.

## Data Availability

The original contributions presented in the study are included in the article/supplementary material, further inquiries can be directed to the corresponding author.
